# Effectiveness and Safety of Bleomycin Electrosclerotherapy for Slow-Flow Vascular Malformations: A Systematic Review

**DOI:** 10.1007/s00270-026-04441-3

**Published:** 2026-04-27

**Authors:** Johannes Gehr, Louise Hindsø, Mikkel Taudorf, Jonas Peter Eiberg, Susanne Christiansen Frevert, Ruben Juhl Jensen, Mikkel Kaltoft, Lars Birger Lönn, Louise Rosenørn de la Motte, Michael Strøm, Caroline Clausen

**Affiliations:** 1 Department of Diagnostic Radiology, Centre of Diagnostic Investigation, Copenhagen University Hospital – Rigshospitalet, Copenhagen, Denmark; 2 Department of Clinical Medicine, Faculty of Health and Medical Sciences, University of Copenhagen, Copenhagen, Denmark; 3 Department of Vascular Surgery, The Heart Centre, Copenhagen University Hospital – Rigshospitalet, Copenhagen, Denmark; 4 Copenhagen Academy for Medical Education and Simulation (CAMES), Copenhagen, Denmark; 5 Department of Otorhinolaryngology, Head and Neck Surgery & Audiology, Centre of Head and Orthopaedics, Copenhagen University Hospital – Rigshospitalet, Copenhagen, Denmark

**Keywords:** Interventional radiology, Bleomycin, Sclerotherapy, Electroporation, Vascular malformations, Congenital

## Abstract

**Purpose:**

To review the available evidence on the effectiveness and safety of bleomycin electrosclerotherapy (BEST) for slow-flow vascular malformations.

**Materials and Methods:**

A systematic review was conducted according to PRISMA guidelines, with a protocol registered in PROSPERO. Five databases were searched, supplemented by citation tracking, to identify peer-reviewed studies reporting clinical outcomes of BEST for slow-flow vascular malformations. Single-patient case reports were excluded. Two authors independently extracted data and assessed risk of bias.

**Results:**

Ten studies were included, with a total of 401 patients and 416 lesions. Seven studies were retrospective, three were prospective, and one was comparative. Any symptom improvement was reported in 62–100% of patients; however, outcome definitions and assessment methods varied substantially across studies, limiting direct comparability. Any size reduction was reported in 83–100% of lesions, based on clinical and imaging-based assessments, including volumetry. Serious adverse events were uncommon, and no systemic toxicity was reported. All non-randomised studies had serious risk of bias.

**Conclusion:**

BEST appears promising for selected slow-flow vascular malformations. However, evidence is limited by methodological heterogeneity and high risk of bias, and treatment decisions should remain individualised and based on multidisciplinary evaluation.

**Graphical Abstract:**

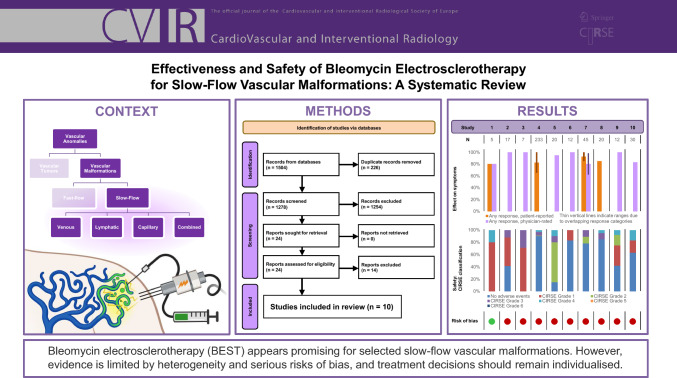

**Supplementary Information:**

The online version contains supplementary material available at 10.1007/s00270-026-04441-3.

## Introduction

Slow-flow vascular malformations (SFVMs) are congenital vascular anomalies that include venous, lymphatic, capillary, and combined lesions. The prevalence of SFVMs, excluding capillary lesions, is estimated at approximately 1 per 1000 live births (0.1%) [1]. Estimates for capillary malformations vary by subtype, with port wine malformations reported in 0.09% of live births [2]. Clinical presentation varies, ranging from cosmetic concerns to pain and functional impairment. For venous and lymphatic malformations, conventional sclerotherapy is regarded as the primary treatment option. Although many patients experience improvement, responses vary widely depending on lesion characteristics and treatment approach, and repeated procedures are often required [3–5].

Bleomycin electrosclerotherapy (BEST) has emerged as a promising treatment approach for SFVMs. The technique is adapted from electrochemotherapy, where electroporation enhances intracellular delivery of poorly permeable agents. In BEST, bleomycin is administered intralesionally and/or intravenously, followed by electroporation to increase its cytotoxicity. The first clinical use of BEST for SFVMs was reported in a 2017 case report by McMorrow et al. In this case, BEST was performed to reduce bleomycin exposure in a patient with pulmonary comorbidity [6]. Since then, its use has expanded, as reflected in a recently published Current Operating Procedure (COP), which indicates its use in cases with poor response or recurrence after previous treatment [7].

To date, only one review has examined electrosclerotherapy for vascular anomalies [8]. However, its scope covered unrelated techniques, such as electrochemical therapy, as well as all vascular anomalies, including vascular tumours. Within that review, included studies predominantly evaluated BEST in patients with SFVMs, consistent with its current clinical use and supporting a focused evaluation in this subgroup. This systematic review aimed to identify, appraise, and synthesise the available literature on the effectiveness and safety of BEST for SFVMs, focusing on symptom improvement, radiological response, and adverse events.

## Materials and Methods

This systematic review was conducted in accordance with the Preferred Reporting Items for Systematic Reviews and Meta-Analyses (PRISMA) guidelines [9]. The protocol was registered in PROSPERO (ID CRD420250651407) in March 2025.

### Information Sources and Search Strategy

Searches were conducted in MEDLINE, EMBASE, Scopus, Web of Science, and the Cochrane Library. The initial search was performed on April 3, 2025, covering all available records. The final search was run on November 4, 2025. No filters were applied.

The search strategy was developed in collaboration with an information specialist and reviewed by all authors. In MEDLINE and the Cochrane Library, we applied the MeSH terms *vascular malformations*, *lymphatic vessels*, *capillaries*, *blood vessels*, *veins*, *port-wine stain*, and *electroporation*. In EMBASE, we used corresponding Emtree terms: *congenital blood vessel malformation*, *lymph vessel*, *capillary*, *blood vessel*, *vein*, *nevus flammeus*, and *electroporation*. Free-text searches were included across all databases to ensure comprehensive coverage. In addition, we performed backward and forward citation tracking of all included studies. Full search strategies are provided in the supplement.

### Eligibility Criteria and Study Selection

Peer-reviewed studies reporting original data on clinical outcomes of BEST for SFVMs in humans of any age were eligible for inclusion. All study designs were eligible, except single-patient case reports. No restrictions were applied regarding publication year or language.

Two authors independently screened all titles and abstracts, followed by full-text assessments of potentially eligible studies. Disagreements were resolved by consensus. Screening and review were conducted using Covidence.

### Outcome Measures and Data Extraction

Data were extracted on study characteristics, patient and lesion characteristics, procedural parameters, effectiveness outcomes, and safety outcomes.

Effectiveness outcomes included changes in symptoms and lesion size. Symptom change was defined as change in any symptom as reported in each study. This was categorised as patient-reported when studies used patient-reported questionnaires and otherwise as physician-assessed. Lesion size change was assessed as any reduction and as relative volume reduction on MRI.

Safety outcomes included the occurrence and severity of adverse events. Adverse event severity was categorised according to the modified Cardiovascular and Interventional Radiological Society of Europe (CIRSE) classification system [10]. For studies using this system, data were extracted as reported. For studies using other classification systems or custom categorisation, reported adverse events were reclassified accordingly.

Two authors independently extracted and classified outcome data. Disagreements were resolved by consensus.

### Data Synthesis

Meta-analysis or other quantitative synthesis was deemed inappropriate due to substantial heterogeneity in study designs, populations, outcome definitions, and reporting. Baseline and procedural characteristics were summarised quantitatively, while effectiveness and safety outcomes were summarised narratively.

### Study Appraisal

For the assessment of internal validity, the Cochrane risk-of-bias tool for randomised trials 2 (RoB2) [11] and the Risk of Bias in Non-randomised Studies of Interventions (ROBINS-I) tool were used [12]. Two authors independently assessed each study, and disagreements were resolved by consensus. The online visualisation tool Robvis was used to present consensus assessments graphically [13].

## Results

### Study Selection

**Fig. 1:**
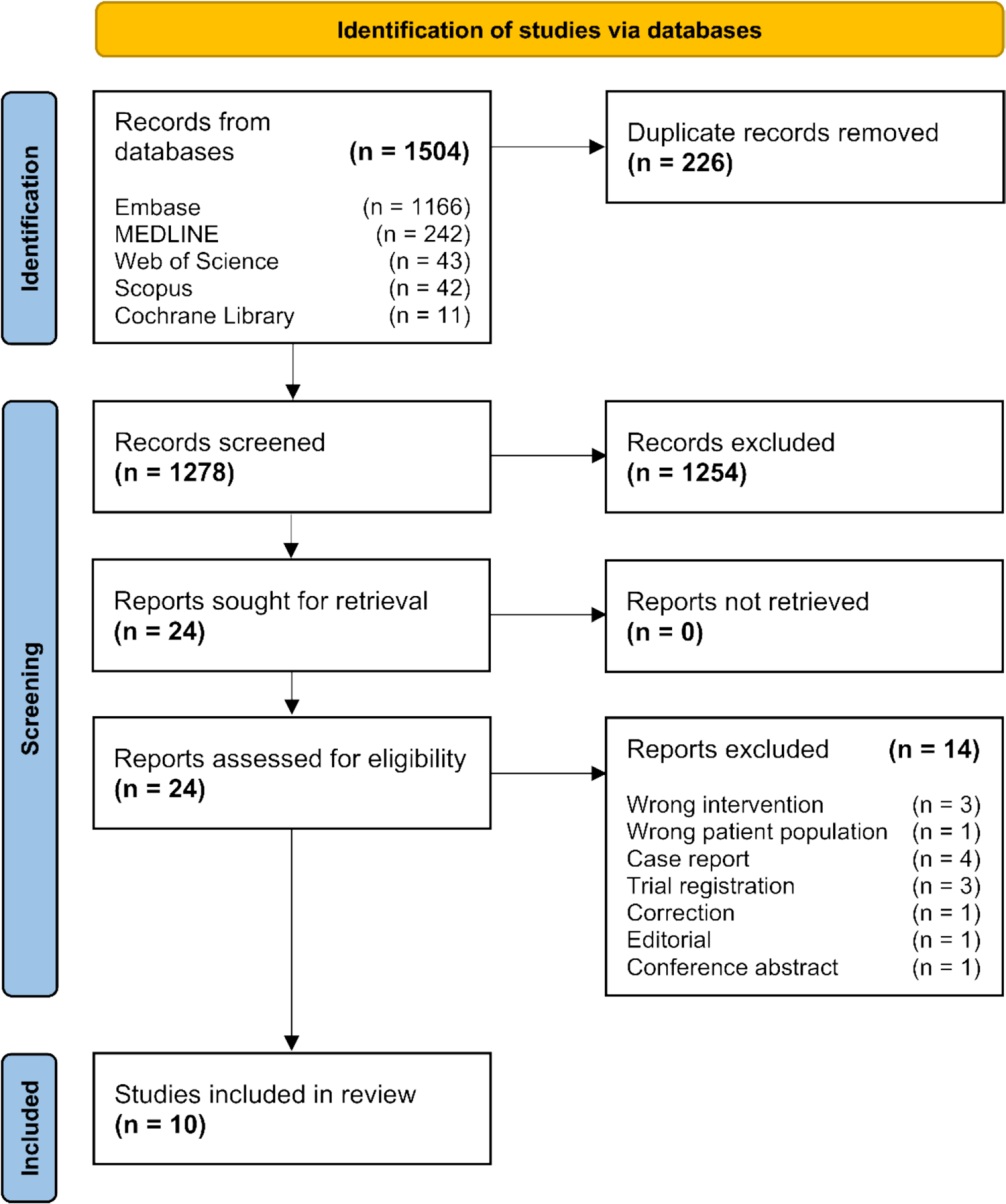
Preferred Reporting Items for Systematic Reviews and Meta–Analyses (PRISMA) flow diagram

The study selection process is presented in the PRISMA flow diagram in Fig. 1. A total of 1504 records were identified. After automatic deduplication, 1278 unique records were screened by title and abstract, of which 24 were retrieved for full-text assessment. Ten studies met all eligibility criteria and were included in this review [14–23].

### Study, Patient, and Procedural Characteristics

A summary of study, patient, and procedural characteristics is presented in Tables 1 and 2.

**Table 1:** Study and population characteristics

		Study					Population		
Author	Year	Country	Design	Patients / lesions	Female, n (%)	Age, years	ISSVA subtypes	Anatomical locations	Previous invasive or targeted therapy, n (%)
Horbach [14]	2020	The Netherlands	RCT	5 / 5	2 (40%)	Median: 64 (36–73)	CM	Head/neck, UE, trunk	0 (0%) *
Wohlgemut [15]	2021	Germany	Retrospective	17 / 20	9 (53%)	Mean: 20.8 ± 8.2	VM	UE, LE, trunk	17 (100%)
Guntau [16]	2023	Germany	Retrospective	7 / 7	3 (43%)	Median: 6 (2–14)	VM, LM, LVM	Tongue	6 (86%)
Schmidt [17]	2024	Germany	Retrospective	233 / 233	115 (49%)	Median: 13 (0–71)	VM, LM, LVM, CVM, CLM, CLVM	Head/neck, UE, LE, trunk	46 (20%)
Vielsmeier [18]	2025	Germany	Retrospective	20 / 20	13 (65%)	Median: 39 (4–78)	VM, LM, LVM	Head/neck, tongue	8 (40%)
Guida [19]	2025	Italy	Prospective	12 / 12	8 (67%)	Median: 6.8 (–IQR 3.8–10.7)	VM, LM, LVM	Head/neck, tongue, UE, LE, trunk	2 (17%)
Haehl [20]	2025	Germany	Retrospective	45 / 45	22 (49%)	Median: 6.5 (0–17) †	VM, LM, LVM, CVM, CLVM	Head/neck, UE, LE, trunk	34 (76%)
Obereisenbuchner [21]	2025	Germany	Retrospective	20 / 20	10 (50%)	Median: 12.5 (2–53)	LM	Head/neck, UE, LE, trunk	14 (70%)
Loeser [22]	2025	Germany	Retrospective	12 / 24	5 (42%)	Mean: 14.8 (range: 0–35)	LM, LVM	Head/neck, tongue, UE, LE, trunk	6 (50%)
Goldann [23]	2025	Germany	Prospective	30 / 30 ‡	10 (42%)	Median: 9.5 (1–17)	VM, LVM, CLVM	Not reported	18 (75%)

^*^ Regions of interest (ROIs) were treatment-naive, while other areas in the same lesions had been treated previously. † Inconsistency: Table 1 reports 8.0 (0–17), while the text reports 6.5 (0–17). ‡ Baseline data is reported only for the 24 patients who attended follow-up. *ISSVA*: International Society for the Study of Vascular Anomalies, *VM*: venous malformation, *LM*: lymphatic malformation, *CM*: capillary malformation, *LVM*: lymphatic-venous malformation, *CVM*: capillary-venous malformation, *CLM*: capillary-lymphatic malformation, *CLVM*: capillary -lymphatic-venous malformation, *UE*: upper extremity, *LE*: lower extremity

**Table 2:** Procedural and outcome characteristics

	Procedure					Outcome		
Author	BEST sessions per treated lesion	Bleomycin administration, sessions (%)	Dose of intralesional bleomycin per session, IU *	Applications per session	Electrode types	Outcome measures	FU, months	Lost to FU, n (%)
Horbach [14]	1	IL: 5/5 (100%)	560	Not reported	Linear needle, plate	GAC and POSAS (patient- and physician-rated scales); LSCI; colorimetry; AEs (no classification)	1.4–1.8	All outcomes: 0
Wohlgemut [15]	Mean: 1.1	IL: 22/22 (100%)	Median: 3000 (1000–7500)	Not reported	Linear needle, hexagonal needle, freely positionable needle, finger	Symptoms (physician-rated); MRI volumetrics; AEs (CIRSE)	Median: 3.7 (3–8.2)	All outcomes: 0
Guntau [16]	Mean: 1.1	Not reported	Not reported	Not reported	Not reported	Symptoms (physician-rated); MRI volumetrics; AEs (no classification)	Median: 16 (IQR 7–35.5)	All outcomes: 0
Schmidt [17]	Mean: 1.4 ± 0.7	IL: 308/325 (95%); IV: 32/325 (10%); Both: 10/325 (3%)	Median: 3000 (500–25,000)	Median: 30 (1–245)	Hexagonal needle, freely positionable needle, finger	Symptoms (patient-rated questionnaire), AEs (CIRSE)	3–12	Questionnaire: 120 (51.5%)
Vielsmeier [18]	Mean: 1.5 ± 0.7	IL: 28/29 (97%); IV: 1/29 (3%)	Mean: 5700 ± 4200	Mean: 12.7 ± 13.7	Finger, stinger	Symptoms (physician-rated); size change (photography/MRI); AEs (no classification)	Median: 3.3 (0.2–12.1)	All outcomes: 0
Guida [19]	Mean: 1.7	IL: 16/20 (80%); IV: 4/20 (20%)	Not reported	Not reported	Not reported	Symptoms (physician-rated); AEs (no classification)	5–6	All outcomes: 0
Haehl [20]	Mean: 1.5 ± 0.8	IL: 64/68 (94%); IV: 2/68 (3%); Both: 2/68 (3%)	Mean: 5900 ± 3,900	Median: 20 (4–141)	Hexagonal, finger	Symptoms (physician-rated and patient-rated questionnaire); AEs (Clavien-Dindo)	3–6	Questionnaire: 4 (8.9%)
Obereisenbuchner [21]	Mean: 1.4 ± 0.6	Not reported	Median: 7000 (2000–15,000)	Median: 26 (6–110)	Hexagonal, finger	Symptoms and QoL (patient-rated questionnaire); MRI volumetrics; AEs (CIRSE)	Mean: 14.3 ± 9.3	QoL: 3 (15%), MRI: 6 (30%),
Loeser [22]	Not reported	Not reported	Mean: 3650 (range: 500–15,000)	Mean: 23.7 (range: 2–82)	Linear needle, hexagonal needle, freely positionable needle, finger	Symptoms (physician-rated); MRI volumetrics; AEs (CIRSE)	Mean: 8.4 (range: 3–20)	All outcomes: 0
Goldann [23]	Mean: 1.8	Not reported	Mean: 3860 ± 2610	Mean: 26.2 ± 31.1	Linear needle, hexagonal needle, freely positionable needle, finger	Symptoms (physician-rated); MRI volumetrics; AEs (no classification)	Mean: 25	All outcomes: 6 (20%)

^*^ Bleomycin doses converted from mg using 1 mg = 1,000 IU. *BEST*: bleomycin electrosclerotherapy, *IU*: international units, *FU*: follow-up, *IL*: intralesional, *IV*: intravenous, *GAC*: global assessment of change, *POSAS*: Patient and Observer Scar Assessment Scale, *LSCI*: laser speckle contrast imaging, *AEs*: adverse events, *CIRSE*: Cardiovascular and Interventional Radiological Society of Europe

#### Study Characteristics

The included studies were published between 2020 and 2025, and all originated from Europe. All were published in English. One study was an RCT, two were prospective non-comparative cohort studies, and seven were retrospective non-comparative cohort studies. Sample sizes ranged from five to 233 participants, and across all studies, a total of 401 patients with 416 lesions were included.

Reported mean or median follow-up ranged from 1.6 to 25 months, with an overall follow-up span from 5 days to 27 months. Outcomes were predominantly reported at variable follow-up intervals, while only two studies used predefined assessment time points [14, 19]. Effectiveness was generally assessed at the last available follow-up, and adverse events were reported from the peri-interventional period throughout follow-up.

#### Patient Characteristics

Across studies, patient age ranged from 10 days to 78 years, nine studies included paediatric participants, and 49% were female patients.

**Fig. 2:**
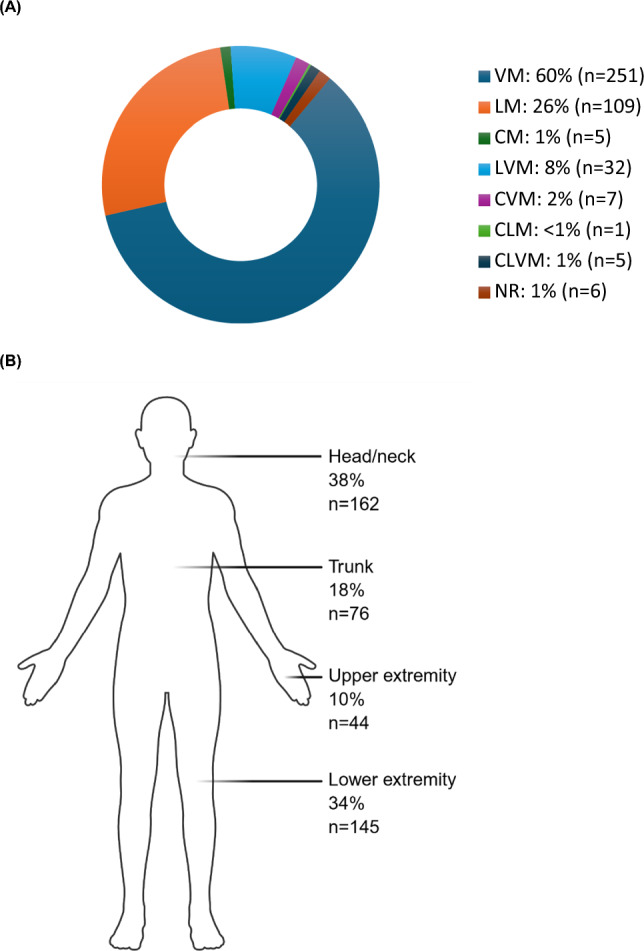
Distribution of vascular malformations across included studies. **A** Donut chart showing cumulative subtype distribution of lesions. VM, venous malformation; LM, lymphatic malformation; CM; capillary malformation; LVM, lymphatic–venous malformation; CVM, capillary–venous malformation; CLM, capillary–lymphatic malformation; CLVM, capillary–lymphatic–venous malformation; NR, not reported. **B** Schematic showing the anatomical distribution of lesions. Goldann et al. [23] lacked anatomical data. In Schmidt et al. [17] and Obereisenbuchner et al. [21], single lesions spanning multiple anatomical regions were counted once per involved region, which leads to a higher summed count (n = 427) than the total number of unique lesions (n = 416). Created with BioRender

Venous malformations were the most frequent ISSVA subtype (61%), followed by lymphatic (27%) and combined malformations (12%) (Fig. 2A). Most studies included multiple subtypes, while four studies [14, 15, 21, 22] focused on single subtypes.

Lesions most commonly involved head and neck (38%) and lower extremities (34%), followed by the trunk (18%) and upper extremities (10%) (Fig. 2B). Two studies [16, 18] included only head and neck lesions, while the remaining reported mixed anatomical locations. Lesion morphology and infiltration were reported systematically only by Haehl et al. [20].

Overall, 38% of patients had undergone previous treatment, most commonly conventional sclerotherapy or surgery. One study [15] included only previously treated lesions, while the remaining studies reported variable proportions (0–76%).

#### Procedural Characteristics

Across studies, the mean number of BEST sessions per lesion ranged from 1.0 to 1.81. Intervals between sessions were reported only in the study by Haehl et al. [20].

Six studies [14, 15, 17–20] reported the route of bleomycin administration, and all used intralesional delivery. Four of these [17–20] also used the intravenous route, accounting for 3–20% of BEST sessions in individual studies. Bleomycin doses were reported in eight studies [14, 15, 17, 18, 20–23], but reporting was inconsistent regarding the use of means and medians. Reported intralesional doses ranged from 560 to 25,000 IU per session across studies.

Seven studies [14, 15, 17–20, 23] used a Cliniporator™ electroporation system (IGEA S.p.A., Carpi, Italy), while the remaining studies did not report on electroporation systems. Electroporations per treatment were reported in six studies [17, 18, 20–23], but reporting formats were inconsistent regarding the use of means and medians. Reported electroporations per treatment ranged from one to 245 across studies.

### Effectiveness Outcomes

Effectiveness outcomes are summarised in Table 3. Reporting was inconsistent, and no study used outcome measures validated for vascular malformations. Six studies [15, 16, 18, 21–23] included MRI-based assessments; five reported volumetric data [15, 16, 21–23]. Four studies [14, 17, 20, 21] used study-specific patient questionnaires. One study [14] used a standardised clinical instrument (The Patient and Observer Scar Assessment Scale (POSAS) [24]), while the remaining studies reported physician-assessed symptom response either narratively or by study-specific scales.

**Table 3:** Summary of improvement in symptoms and reduction in size after BEST across included studies

		Improvement in symptoms				Reduction in size	
Study	N	Patient-reported		Physician-assessed		Lesions with any reduction in size	Volume reduction on MRI
		Any response	Complete response	Any response	Complete response		
Horbach [14]	5	80%	20%	80%	0%	–	–
Wohlgemut [15]	17	–	–	100%	47%	95% (n = 20)	Median: 86% (n = 20)
Guntau [16]	7	–	–	100%	–	100% (n = 5)	Median: 39% (n = 5)
Schmidt [17]	233	65–100%* (n = 113)	0–10%*† (n = 113)	–	–	–	–
Vielsmeier [18]	20	–	–	95%	–	95%	–
Guida [19]	12	–	–	100% (n = 8)	75% (n = 8)	–	–
Haehl [20]	45	85–100%* (n = 41)	0–24%*† (n = 41)	62–98%*	–	–	–
Obereisenbuchner [21]	20	85%	35%	–	–	–	Change in mean: 31% (n = 14)
Loeser [22]	12	–	–	100%	0%	83% (n = 24)	Mean: 55% (n = 24)
Goldann [23]	30	–	–	83% (n = 24)	8% (n = 24)	100% (n = 24)	Median: 59% (n = 24)

Any response’ and ‘complete response’ denote symptom improvement as reported in each study. ‘Complete response’ is a subset of ‘any response’; both proportions are calculated from the total cohort (N) or from the relevant subgroup or lesion count (n). Unavailable data are indicated by “–“. * Range reflects uncertainty due to non–paired reporting without information on patient overlap. † Outcome assessed across multiple domains; complete response required asymptomatic status or equivalent score across all domains. *BEST*: bleomycin electrosclerotherapy

Because of heterogeneity in outcome definitions, symptom outcomes were categorised as any response and complete response, as defined in each study. Reported symptoms varied across studies and included pain, swelling, functional impairment, aesthetic concerns, and site-specific manifestations. Lesion size outcomes were summarised as any size reduction and MRI-based volumetric change, as measured in each study.

Across physician-assessed outcomes, any symptom improvement was reported in ≥ 80% of patients in seven of the eight studies reporting this outcome (range 62–100%). Complete response was reported in five studies, with rates ≤ 47% in four studies and 75% in one (range 0–75%). Notably, the largest included study [17] did not report physician-assessed outcomes. In the four studies using patient-reported questionnaires [14, 17, 20, 21], any improvement ranged from 65 to 100%, and complete response from 0 to 35%. Five studies [15, 16, 18, 22, 23] reported lesion-level data on size reduction, with any reduction observed in 83–100% of evaluated lesions, based on either MRI volumetry or clinical assessment. Five studies [15, 16, 21–23] reported MRI-based relative volume reduction ranging from 31 to 86%, reported as either means or medians. Volumetry was performed mainly using geometric approximations, and in one, study manual segmentation [16].

### Safety Outcomes

Safety outcomes are presented in Table 4.

**Table 4:** Adverse events classified according to the modified Cardiovascular and Interventional Radiological Society of Europe (CIRSE) classification across included studies

Study	N	No adverse events	CIRSE grade 1		CIRSE grade 2	CIRSE grade 3		CIRSE grade 4	CIRSE grade 5	CIRSE grade 6
			1a	1b		3a	3b			
Horbach [14] *	5	0%	80%	0%	0%	0%		20%	0%	0%
Wohlgemut [15]	17	41%	47%	0%	0%	12%		0%	0%	0%
Guntau [16] *	7	0%	71%	0%	0%	29%		0%	0%	0%
Schmidt [17] †‡	233	90%	0% (1/325)	0%	1%	6%		3%	0%	0%
Vielsmeier [18] *§	20	15%	0%		65%	10%		10%	0%	0%
Guida [19] *	12	83%	17%	0%	0%	0%		0%	0%	0%
Haehl [20] *†	45	78%	0%		11%	11%		0%	0%	0%
Obereisenbuchner [21]	20	85%	0%		0%	10%		5%	0%	0%
Loeser [22] *	12	42%	33%	0%	17%	0%		8%	0%	0%
Goldann [23] *	30	63%	20%	0%	0%	17%		0%	0%	0%

Percentages represent the proportion of patients within each individual study. For each patient, only the highest CIRSE grade is counted, irrespective of the number of treatment sessions or lesions treated, unless otherwise specified. CIRSE subgrades (1a/b and 3a/b) are shown only when distinguishable from the information provided. * CIRSE classifications were absent or incomplete; grades were assigned based on the reported adverse event details. † Skin discoloration was patient–reported only and was not included as an adverse event. ‡ Adverse events were reported per procedure (n = 325), as patient–level data were unavailable. § Adverse events were reported in 17/20 patients; only four events could be graded (CIRSE 3–4), while the remaining events were conservatively classified as grade 2 due to lack of patient–level reporting

All studies reported on adverse events. Four studies [15, 17, 21, 22] applied the original CIRSE classification [25], one study [20] used the Clavien-Dindo system [26], and the remaining five studies reported narratively or through study-specific categories. No study applied the modified CIRSE classification.

No study reported systemic bleomycin-related toxicity, pulmonary complications, or allergic reactions.

No Grade 5 or 6 events were identified after recoding according to the modified CIRSE classification. Grade 4 events occurred in five studies [14, 17, 18, 21, 22], with rates up to 20% in the smallest study [14]. The most common Grade 4 events were hypertrophic scarring and persistent nerve injury, both described as long-term sequelae. Pharyngeal oedema requiring tracheostomy was reported as an acute event and classified as Grade 4 based on clinical severity.

Grade 3 events were reported in several studies, but in low numbers. They included infections, ulceration, and bleeding, with reported rates generally below 20%.

Most patients experienced either no adverse events or Grade 1a events. Grade 1a events included local reactions such as pain, swelling, and erythema (reported duration range: < 1–12 weeks), and temporary or persistent local skin discolouration (reported duration range: 3–24 months). No Grade 1b events were identified. Vielsmeier et al. [18] reported several events with insufficient detail for classification; these were conservatively assigned as Grade 2.

### Risk of Bias

**Fig. 3:**
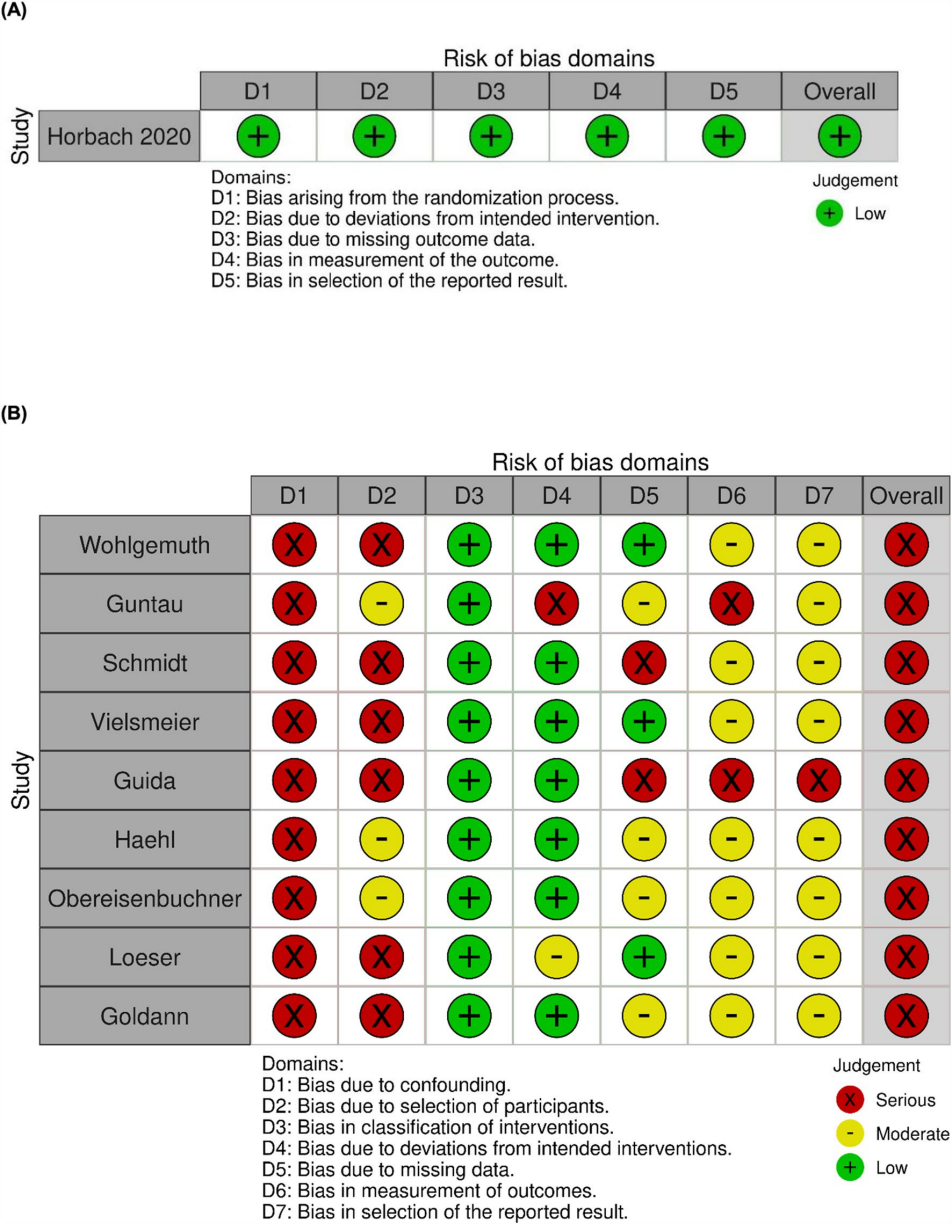
Risk of bias assessment of included studies visualised using Robvis. **A** Horbach et al. [14], assessed using the Cochrane Risk of Bias 2 tool (RoB 2). **B** Remaining studies, assessed using the Risk Of Bias In Non–randomized Studies of Interventions tool (ROBINS–I)

Risk-of-bias assessments are shown in Fig. 3. All nine non-randomised studies were judged to have a serious overall risk of bias, mainly because of confounding. All were single-arm studies with incompletely reported baseline characteristics, and none accounted for prognostic factors in their analyses. Participant selection also raised concerns, as referral pathways, indications, and inclusion processes were often insufficiently described.

Most of these studies used non-blinded, non-standardised, and non-validated assessments. Missing data were described narratively, and no study used predefined strategies for handling incomplete follow-up. In the largest study [17], outcome data were missing in more than half of the patients. Selective reporting could not be excluded because none of the studies had a preregistered protocol.

Classification of interventions and adherence to intended interventions were consistently well described and judged at low risk. The only RCT [14] was judged at low risk across all domains.

## Discussion

Across the included studies, BEST was generally associated with symptomatic improvement and reductions in lesion size in patients with SFVMs. The reported safety profile was favourable, as most adverse events were low-grade and no systemic toxicity was observed. These findings should nevertheless be interpreted with caution, as the evidence remains uncertain due to methodological limitations and heterogeneity in reporting.

The available evidence provides initial insight into the emerging role of BEST in the management of SFVMs. Within the included studies, the technique was applied to a range of lesion types, reflecting the evolving clinical indications. Notably, several studies included lesions that often respond poorly to conventional treatments, such as mucosal head and neck lesions, microcystic lymphatic malformations, and large or infiltrative lesions, yet reported favourable outcomes. Only one study [15] restricted inclusion to previously treated lesions, in line with the COP indication for treatment-resistant cases. In relation to this, two studies [17, 21] examined whether prior treatment influenced outcomes, but reported conflicting results, likely due to small sample sizes. Collectively, these observations suggest that BEST may expand therapeutic options in settings where existing treatment approaches are limited, while its potential role in treatment-naive lesions remains less well characterised.

However, interpretation of the available evidence is constrained by several methodological limitations. Most included studies were small observational cohorts, often retrospective and non-comparative, limiting attribution of observed effects to BEST. In addition, marked heterogeneity in outcome definitions, reporting formats, and procedural parameters complicates comparison of results between studies. Confounding by indication remains a concern, as treatment selection was often not clearly described. Furthermore, most studies originated from a small number of centres, predominantly in Germany, which may influence patterns of clinical use and limit the generalisability of the findings. Finally, harmonisation of effectiveness and adverse event data required reclassification of outcomes across studies, introducing a risk of misclassification bias.

Considering these limitations, future research should prioritise standardised outcome measures, consistent reporting of procedural parameters, and clearly defined cohorts. This will enable more reliable characterisation of treatment effects and safety, and facilitate comparisons between studies. The use of validated condition-specific instruments, such as the Outcome Measure for Vascular Anomalies (OVAMA) [27], may improve the consistency and clinical interpretability of reported outcomes. In particular, outcomes should be analysed according to clinically relevant lesion and patient characteristics, such as ISSVA subtype, anatomical location, or lesion depth. Such knowledge will be essential for designing meaningful comparative studies to determine whether and when BEST offers advantages over conventional treatments.

## Conclusion

BEST appears promising for selected SFVMs, with consistent reports of symptomatic improvement, lesion reduction, and a favourable safety profile across studies. However, small sample sizes, retrospective designs, lack of comparators, and outcome heterogeneity weaken the evidence and limit its reliability. Treatment decisions should therefore remain individualised and based on multidisciplinary evaluation. Future studies should use validated outcome measures and clearly defined cohorts to enable meaningful comparative research.

## Supplementary Information

Below is the link to the electronic supplementary material.

Supplementary file1 (DOCX 108 KB)
