# Supplementary material for: Effectiveness and Safety of Bleomycin Electrosclerotherapy for Slow-Flow Vascular Malformations: A Systematic Review
Source: Cardiovasc Intervent Radiol. 2026 Apr 27;49(6):1105–16. doi: 10.1007/s00270-026-04441-3 (PMC13212693; doi:10.1007/s00270-026-04441-3)
Supplement: Supplementary file 1 — Supplementary file1 (DOCX 108 KB) [file 270_2026_4441_MOESM1_ESM.docx]

**FULL DATABASE SEARCHES**

No limits were applied. See the main article for search dates and numbers of records retrieved.

| **MEDLINE (via Ovid, 1966 to present)** | |
| --- | --- |
|  | **Search terms** |
| #1 | exp Vascular Malformations/ |
| #2 | exp Lymphatic Vessels/ |
| #3 | exp Capillaries/ |
| #4 | exp Blood Vessels/ |
| #5 | exp Veins/ |
| #6 | exp port-wine stain/ |
| #7 | ((vascul* or vessel* or venous or vein* or capillar* or lymph* or slow-flow or low-flow or venolymphatic) adj10 (malform* or abnorm* or anomal* or lesion* or disorder* or dysplas* or patholog*)).af. |
| #8 | (vascul* birthmark* or vascul* birth mark* or port-wine stain* or portwine stain* or n?evus flammeus).af. |
| #9 | FAVA.af. |
| #10 | #1 or #2 or #3 or #4 or #5 or #6 or #7 or #8 or #9 |
| #11 | exp Electroporation/ |
| #12 | (bleomycin* and (electrochemotherap* or electro-chemotherap* or electrosclerotherap* or electro-sclerotherap* or electroporation* or electro-poration* or electropermeab* or electro-permeab* or electrotransfer* or electro-transfer* or electropulsation* or electro-pulsation*)).af. |
| #13 | #11 or #12 |
| #14 | #10 and #13 |

| **EMBASE (via Ovid, 1974 to present)** | |
| --- | --- |
|  | **Search terms** |
| #1 | exp congenital blood vessel malformation/ |
| #2 | exp lymph vessel/ |
| #3 | exp capillary/ |
| #4 | exp blood vessel/ |
| #5 | exp vein/ |
| #6 | exp nevus flammeus/ |
| #7 | ((vascul* or vessel* or venous or vein* or capillar* or lymph* or slow-flow or low-flow or venolymphatic) adj10 (malform* or abnorm* or anomal* or lesion* or disorder* or dysplas* or patholog*)).af. |
| #8 | (vascul* birthmark* or vascul* birth mark* or port-wine stain* or portwine stain* or n?evus flammeus).af. |
| #9 | FAVA.af. |
| #10 | #1 or #2 or #3 or #4 or #5 or #6 or #7 or #8 or #9 |
| #11 | exp electroporation/ |
| #12 | (bleomycin* and (electrochemotherap* or electro-chemotherap* or electrosclerotherap* or electro-sclerotherap* or electroporation* or electro-poration* or electropermeab* or electro-permeab* or electrotransfer* or electro-transfer* or electropulsation* or electro-pulsation*)).af. |
| #13 | #11 or #12 |
| #14 | #10 and #13 |

| **The COCHRANE Library** | |
| --- | --- |
|  | **Search terms** |
| #1 | MeSH descriptor: [Vascular Malformations] explode all trees |
| #2 | MeSH descriptor: [Lymphatic Vessels] explode all trees |
| #3 | MeSH descriptor: [Capillaries] explode all trees |
| #4 | MeSH descriptor: [Blood Vessels] explode all trees |
| #5 | MeSH descriptor: [Veins] explode all trees |
| #6 | MeSH descriptor: [Port-Wine Stain] explode all trees |
| #7 | ((vascul* or vessel* or venous or vein* or capillar* or lymph* or slow-flow or low-flow or venolymphatic) NEAR/10 (malform* or abnorm* or anomal* or lesion* or disorder* or dysplas* or patholog*)) |
| #8 | (vascul* NEXT birthmark* or vascul* NEXT birth NEXT mark* or port-wine NEXT stain* or portwine NEXT stain* or n?evus NEXT flammeus) |
| #9 | FAVA |
| #10 | #1 or #2 or #3 or #4 or #5 or #6 or #7 or #8 or #9 |
| #11 | MeSH descriptor: [Electroporation] explode all trees |
| #12 | (bleomycin* and (electrochemotherap* or electro-chemotherap* or electrosclerotherap* or electro-sclerotherap* or electroporation* or electro-poration* or electropermeab* or electro-permeab* or electrotransfer* or electro-transfer* or electropulsation* or electro-pulsation*)) |
| #13 | #11 or #12 |
| #14 | #10 and #13 |

| **Web of Science** | |
| --- | --- |
|  | **Search terms** |
| #1 | TS=((vascul* or vessel* or venous or vein* or capillar* or lymph* or slow-flow or low-flow or venolymphatic) NEAR/10 (malform* or abnorm* or anomal* or lesion* or disorder* or dysplas* or patholog*)) |
| #2 | TS=(("vascul* birthmark*" or "vascul* birth mark*" or "port-wine stain*" or "portwine stain*" or "n$evus flammeus")) |
| #3 | TS=(FAVA) |
| #4 | TS=((bleomycin* and (electrochemotherap* or electro-chemotherap* or electrosclerotherap* or electro-sclerotherap* or electroporation* or electro-poration* or electropermeab* or electro-permeab* or electrotransfer* or electro-transfer* or electropulsation* or electro-pulsation*))) |
| #5 | #1 OR #2 OR #3 |
| #6 | #4 AND #5 |

| **SCOPUS** | |
| --- | --- |
|  | **Search terms** |
| #1 | TITLE-ABS-KEY(((vascul* OR vessel* OR venous OR vein* OR capillar* OR lymph* OR slow-flow OR low-flow OR venolymphatic) W/10 (malform* OR abnorm* OR anomal* OR lesion* OR disorder* OR dysplas* OR patholog*))) |
| #2 | TITLE-ABS-KEY(("vascul* birthmark*" OR "vascul* birth mark*" OR "port-wine stain*" OR "portwine stain*" OR "n*evus flammeus")) |
| #3 | TITLE-ABS-KEY(FAVA) |
| #4 | TITLE-ABS-KEY((bleomycin* AND (electrochemotherap* OR electro-chemotherap* OR electrosclerotherap* OR electro-sclerotherap* OR electroporation* OR electro-poration* OR electropermeab* OR electro-permeab* OR electrotransfer* OR electro-transfer* OR electropulsation* OR electro-pulsation*))) |
| #5 | #1 OR #2 OR #3 |
| #6 | #4 AND #5 |
